# Uncovering and Engineering a Mini-Regulatory Network of the TetR-Family Regulator SACE_0303 for Yield Improvement of Erythromycin in *Saccharopolyspora erythraea*

**DOI:** 10.3389/fbioe.2021.692901

**Published:** 2021-09-14

**Authors:** Ying Liu, Sabir Khan, Panpan Wu, Bowen Li, Lanlan Liu, Jingshu Ni, Hongxia Zhang, Ketao Chen, Hang Wu, Buchang Zhang

**Affiliations:** School of Life Sciences, Institute of Physical Science and Information Technology, Anhui University, Hefei, China

**Keywords:** TetR-family regulator, erythromycin, MarR-family regulator, *Saccharopolyspora erythraea*, regulatory network, CRISPRi, system metabolic engineering

## Abstract

Erythromycins produced by *Saccharopolyspora erythraea* have broad-spectrum antibacterial activities. Recently, several TetR-family transcriptional regulators (TFRs) were identified to control erythromycin production by multiplex control modes; however, their regulatory network remains poorly understood. In this study, we report a novel TFR, SACE_0303, positively correlated with erythromycin production in *Sac. erythraea*. It directly represses its adjacent gene *SACE_0304* encoding a MarR-family regulator and indirectly stimulates the erythromycin biosynthetic gene *eryAI* and resistance gene *ermE*. SACE_0304 negatively regulates erythromycin biosynthesis by directly inhibiting *SACE_0303* as well as *eryAI* and indirectly repressing *ermE*. Then, the SACE_0303 binding site within the *SACE_0303-SACE_0304* intergenic region was defined. Through genome scanning combined with *in vivo* and *in vitro* experiments, three additional SACE_0303 target genes (*SACE_2467* encoding cation-transporting ATPase, *SACE_3156* encoding a large transcriptional regulator, *SACE_5222* encoding α-ketoglutarate permease) were identified and proved to negatively affect erythromycin production. Finally, by coupling CRISPRi-based repression of those three targets with *SACE_0304* deletion and *SACE_0303* overexpression, we performed stepwise engineering of the SACE_0303-mediated mini-regulatory network in a high-yield strain, resulting in enhanced erythromycin production by 67%. In conclusion, the present study uncovered the regulatory network of a novel TFR for control of erythromycin production and provides a multiplex tactic to facilitate the engineering of industrial actinomycetes for yield improvement of antibiotics.

## Introduction

The high G+C Gram-positive bacterial actinomycetes are well known as one of the most abundant sources of bioactive secondary metabolites (Barka et al., 2016). The biosynthetic gene clusters of antibiotics in actinomycetes are transcriptionally controlled through subtle and delicate regulatory mechanisms, during which cluster-situated regulators (CSRs) or global regulators modulate the expression of their targets by sensing environmental or physiological signals (Niu et al., 2016; van der Heul et al., 2018). Thus far, at least 20 families of transcription factors (TFs) have been found in the antibiotic-producing actinomycetes (Romero-Rodriguez et al., 2015). Engineering of those regulators and their targets that control the biosynthesis of pharmaceutical antibiotics is an effective manner to boost the productivity of these fermentation products (Martín and Liras, 2010).

*Saccharopolyspora erythraea*, an important industrial actinomycete, is commonly used for the large-scale fermentation manufacturing of the valuable polyketide antibiotic erythromycin A (Er-A). Er-A and its derived macrolide drugs exhibit nice activities of many Gram-positive and some Gram-negative bacteria and have high annual sales in the billions of dollars (Robertsen and Musiol-Kroll, 2019). Hence, titer improvement of industrial erythromycin production is of vital significance. The erythromycin biosynthetic gene (*ery*) cluster consists of 20 genes arranged in four main polycistronic units in *Sac. erythraea* (Oliynyk et al., 2007). In general, there is lack of insight into the transcriptional regulation of the *ery* cluster owing to the absence of a CSR gene (Mironov et al., 2004). This not only implicates the unique mechanism for regulating erythromycin biosynthesis, but also increases the difficulty in regulatory engineering of *Sac. erythraea* for erythromycin biosynthetic titer improvement.

The genome of *Sac. erythraea* encodes 1,118 genes with latent regulatory functions, in which numerous types of TFs were discovered (Oliynyk et al., 2007). The developmental regulator BldD was first proved to directly control the synthesis of erythromycin (Chng et al., 2008). Next, several types of TFs were subsequently reported to be involved in erythromycin production in *Sac. erythraea*, mainly including the families of TetR and Lrp as well as nutrient-sensing regulators (Wu et al., 2014a,b, 2016, 2019; Liu et al., 2017, 2019; Xu et al., 2018, 2019b). This uncovered the complicated mechanism for controlling erythromycin biosynthesis with reciprocal (interactive regulation) or cascaded (hierarchical regulation) modes, which enable us to understand and manipulate the regulatory network governing erythromycin biosynthesis for titer improvement.

As a typical representative of TFs, TetR family transcriptional regulators (TFRs), consisting of an N-terminal DNA-binding domain and a C-terminal ligand-responsive domain, usually participate in the control of antibiotic production in actinomycetes (Cuthbertson and Nodwell, 2013). A total of 97 TFRs were encoded by the *Sac. erythraea* genome (Wu et al., 2016), and only five of them (SACE_3986, SACE_7301, SACE_3446, PccD, and SACE_5754) have been successively reported to control the biosynthesis of erythromycin so far (Wu et al., 2014a,b, 2016, 2019; Xu et al., 2018). These investigations show varied molecular mechanisms for regulating erythromycin production (negatively or positively) or the *ery* cluster (directly or indirectly). In particular, little is known about the TFR-mediated regulatory network concerning erythromycin biosynthesis.

Although manipulation of those TFRs and (or) their targets resulted in yield improvement of erythromycin, traditional genetic engineering in *Sac. erythraea* remained limited owing to much time and effort regarding multigene engineering. In particular, the low efficiency of the homologous recombination-based gene knockout in *Sac. erythraea* has always restricted genetic engineering of the industrial actinomycetes. In the past 3 years, clustered regularly interspaced short palindromic repeats interference (CRISPRi) mediated multiplex gene repression has been developed in the model actinomycetes *Streptomyces coelicolor* (Zhao et al., 2018) and was subsequently utilized in the industrial *Streptomyces rapamycinicus* and *Streptomyces bingchenggensis* to improve the titers of rapamycin and milbemycin, respectively (Tian et al., 2020; Liu et al., 2021b).

In this study, we report a novel TFR, SACE_0303, which indirectly triggered the erythromycin structure gene *eryAI* and resistance gene *ermE*, but directly suppressed its adjacent gene *SACE_0304*, encoding a MarR-family regulator (MFR). SACE_0304 was shown to directly repress SACE_0303 and *eryAI* but indirectly inhibit *ermE*. Three new SACE_0303’ target genes, *SACE_2467*, *SACE_5222*, and *SACE_3156*, were discovered and validated to negatively affect erythromycin production. Further, we performed stepwise engineering of the SACE_0303-mediated mini-regulatory network in a high-yield strain by coupling CRISPRi-based repression of those three targets with *SACE_0304* deletion and *SACE_0303* overexpression, resulting in obvious titer improvement of erythromycin.

## Materials and Methods

### Bacterial Strains, Media, Cultivation Conditions, Plasmids, and Primers

All strains, plasmids, and primers used in this study are listed in Supplementary Tables 1–3. *Escherichia coli* were cultured in Luria-Bertani (LB) broth medium or on LB agar plate at 37°C. *E. coli* DH5α were used to construct plasmid. *E. coli* BL21 (DE3) was used for protein expression. *Sac. erythraea* A226, WB, and their derivative mutants were grown on the R3M agar plate medium for sporulation, protoplast regeneration, and phenotypic observation and in tryptone soya broth (TSB) medium for seed stock culture, genomic DNA extraction, and protoplast preparation at 30°C.

### Deletion and (or) Overexpression of SACE_0303 and Its Targets in *Sac. erythraea*

With the genome DNA of A226 as a template, two 1.5-kb DNA fragments flanking the *SACE_0303* gene were amplified via PCR with the primer pairs SACE_0303-up-F/R and SACE_0303-down-F/R, respectively (Supplementary Table 3). The amplified fragments were successively treated with EcoRI/KpnI and XbaI/HindIII and ligated into the corresponding sites of pUCTSR (Han et al., 2011), generating pUCTSR-Δ*0303* (Supplementary Table 2). Plasmid pUCTSR-Δ*0303* was introduced into *Sac. erythraea* A226 by PEG-mediated protoplast transformation. A 424-bp DNA fragment of S*ACE_0303* was replaced by the thiostrepton resistance gene (*tsr*) by the method of chromosomic homologous recombination. The Δ*SACE_0303* mutant with thiostrepton resistance was confirmed by PCR with the primers SACE_0303-C1/C2 (Supplementary Table 3). A full-length *SACE_0303* gene of 564-bp were amplified by PCR using the primers SACE_0303-C1/C2 (Supplementary Table 3) from the genomic DNA of A226. The amplified fragment and pIB139 were cleaved with NdeI/Xbal and ligated to generate pIB139-0303 (Supplementary Table 2). Plasmid pIB139-0303 was introduced into Δ*SACE_0303* and A226, respectively, by PEG-mediated protoplast transformation. The complemented strains Δ*SACE_0303*/pIB-0303 and overexpression strain A226/pIB-0303 were obtained by apramycin resistance screening and confirmed by PCR analysis with primers Apr-test-F/R (Supplementary Table 3). The same genetic manipulation methods were applied to construct *SACE_0304* relevant mutants in A226 with corresponding primers (Supplementary Table 3). Likewise, three additional SACE_0303 target genes were individually overexpressed in A226 with their respective primers, and corresponding strains A226/pIB-2467, A226/pIB-3156, and A226/pIB-5222 were obtained (Supplementary Tables 1–3).

Moreover, *SACE_0303* overexpression and *SACE_0304* deletion in the high-yield *Sac. erythraea* WB were also manipulated, subsequently generating the mutants WB/pIB-0303 and WBΔ*0304* (Supplementary Tables 1, 2).

### Construction of CRISPRi Plasmids and Relevant *Sac. erythraea* Mutants

With the plasmid DNA of pSET-dCas9-*actII4*-NT-S1 as a template, the *ermE*^∗^ promoter was obtained by PCR amplification with the primers P-PermE^∗^-F/R (Supplementary Table 3). With the genomic DNA of A226 as a template, the *SACE_0303* gene was amplified with the primers P0303-F/R (Supplementary Table 3). Then, the two fragments were, respectively, digested with EcoRV/XbaI and XbaI/KpnI and ligated to the EcoRV/KpnI sites of pSET-dCas9-*actII4*-NT-S1 to generate pSETdCas9*-*0303 (Supplementary Table 2). The 20 nt sequences of the genes *SACE_2467*, *SACE_3156*, and *SACE_5222* were obtained by the software sgRNA cas9 (v3.0) (Xie et al., 2014), which is suitable for gene editing with CRISPRi technology operation. A 699 bp sgRNA tandem sequence of the three SACE_0303 target genes synthesized from Sangon Biotech (Supplementary Figure 4) was digested with KpnI/EcoRI and ligated into the corresponding sites of pSETdCas9-0303, generating pSETdCas9-0303-sg2467*-*3156-5222 (Supplementary Table 2). Finally, pSETdCas9-0303 was successively introduced into the WB and WBΔ*0304* to obtain WB/pSETdCas9*-*0303 and WBΔ*0304*/pSETdCas9*-*0303, respectively (Supplementary Table 1), and pSETdCas9-0303-sg2467-3156-5222 was transformed into WBΔ*0304* to generate the strain WBΔ*0304*/p0303-sg2467-3156-5222 (Supplementary Table 1).

### Fermentation and Measurement of Erythromycin

Flask fermentation of A226, WB, and their derived mutants were performed as previously described. Spores of A226 and its derivative strains were inoculated into TSB seed medium and grown for 2 days. Then, 5 mL seed cultures were inoculated into the R5 liquid medium to grow at 220 rpm, 30°C for 6 days. For WB and its derivatives, strains were cultivated in the industrial seed and fermentation media with the same culture conditions as A226 (Wu et al., 2014a). Er-A extracted from those fermentation broths was quantitatively measured by HPLC. An Agilent Extend-C18 column (5 μm; 250 × 4.6 mm) was equipped in the Shimadzu LC-2030 Plus HPLC system equilibrated with 60% solution A (5 mM ammonia acetate, pH 7.0) and 40% solution B (acetonitrile). An isocratic program was performed at a flow rate of 1.0 mL/min at 30°C using a ELSA-LT II ELSD detector (Wu et al., 2014a).

### Protein Expression and Purification

The *SACE_0303* gene was amplified using the primers SACE_0303-C5/C6 (Supplementary Table 3) and was cloned into the NdeI/HindIII sites of pET28a to generate pET28a-0303. pET28a-0303 was transformed into *E. coli* BL21 (DE3), and SACE_0303 expression was induced by 0.5 mM IPTG at 30°C for 8–10 h. Purification of His_6_-tagged SACE_0303 protein was performed on a Ni^2+^-NTA spin column (BIO-RAD). BCA protein assay kit (Thermo Fisher Scientific) was used to analyze the concentration of purified protein, and its quality was estimated by SDS-PAGE.

### Electrophoretic Mobility Shift Assays (EMSAs)

EMSAs were performed as previously published report (Hellman and Fried, 2007). DNA probes were amplified by PCR with their respective primers listed in Supplementary Table 3. The DNA probe was incubated with various concentrations of His_6_-tagged SACE_0303. The binding reaction system contained 60 mM KCl, 50 mM EDTA, 10 mM Tris-HCl (pH 7.5), 10 mM DTT, 5 mM MgCl_2_, 10% glycerol, 150 ng DNA probe labeled by 5′-FAM/3′-HEX and purified His_6_-SACE_0303 protein. Unlabeled DNA fragments or poly-dIdC were used for competitive assays. After incubation at 30°C for 20 min in 20 μL reaction mixtures, the reactants were fractionated on 6% native PAGE gels in 1 × TAE buffer at 40 mA for 35–45 min.

### Real-Time Quantitative PCR (RT-qPCR) Assay

Using the TransZol up plus RNA kit (Transgen), total RNA was isolated from A226 and its derivatives after 24 h fermentation in R5 liquid medium or WB derivatives after 12 h culture in industrial fermentation medium. The RNA concentration was measured with the microplate reader (BioTek). RNA was treated with DNase I (MBI Fermentas), and reverse transcription was achieved using a cDNA synthesis kit (MBI Fermentas). The relative transcriptional levels of genes were examined with QuantStudio^TM^ 6 Flex (Thermo Fisher Scientific) using the primers listed in Supplementary Table 3. The *hrdB* (*SACE_1801*) gene in *Sac. erythraea* was served as an internal control to normalize samples.

### eGFP Reporter Assay

DNA fragments of four promoters containing P_0303_, P_0304_, P*_*eryAI*_*, and P*_*ermE*_* regions were successively amplified using the primer pairs in Supplementary Table 3 with A226 as the template and digested with HindIII/XbaI. The enhanced green fluorescent protein gene (*egfp*) fragment obtained by XbaI/BamHI digestion of pKC-DE (Liu et al., 2017) and the above PCR products were ligated into HindIII/BamHI sites of pKC1139 (Wilkinson et al., 2002) to obtain the control plasmids pKC-ME, pKC-TE, pKC-AE, and pKC-EE (Supplementary Table 2). Next, the P*_*apr*_* with EcoRV/NdeI was obtained from pIB139 using the primers P-P*_*apr*_*-F/R (Supplementary Table 3), and the *SACE_0304* gene with NdeI/EcoRI was amplified using the primers SACE_0304-F-F/R (Supplementary Table 3) with A226 genomic DNA as the template. Then, the two fragments were jointly ligated into EcoRV/EcoRI sites of the above four control plasmids, generating the reporter plasmids pKC-MR-ME, pKC-MR-TE, pKC-MR-AE, and pKC-MR-EE, respectively (Supplementary Table 2).

The above plasmids were separately transformed into DH5α for detection of green fluorescence (excitation at 485 nm; emission at 510 nm, Molecular Devices). All fluorescence values were normalized to growth rates (OD600).

### DNase I Footprinting Assay

The DNase I footprinting assay was performed as previously described (Xu et al., 2019a). To precisely determine the DNA binding site of SACE_0303, 100 ng FAM/HEX-labeled P_*0303–0304*_ was successively incubated with 0, 70, and 490 nM His6-SACE_0303 in a total 50 μL of binding buffer at 20°C for 20 min, and then 2 μL DNase I (1 U/μg; Promega) was performed at 20°C for 30 s, 10 μL DNase I stop solution was added to the mixture and reacted at 65°C for 10 min. The ethanol precipitation method was used to purify and recover DNA samples. Purified DNA was sequenced with a 3730XL DNA genetic analyzer (Applied Biosystems), and GeneMarker software program v2.2 for data analysis.

### Statistical Analyses

All data in this study were stated as means ± standard error of the mean (SD), and analyzed by Student’s *t*-test, with ^∗^*p* <0.05, ^∗∗^ *p* < 0.01, and ^∗∗∗^*p* < 0.001, ns, not significant.

## Results

### SACE_0303 Positively Affects the Erythromycin Production

**FIGURE 1:**
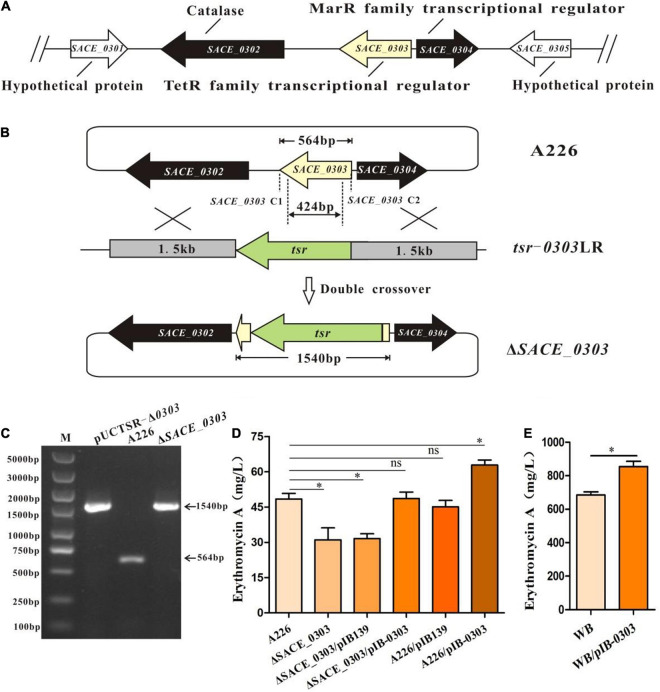
Inactivation of *SACE_0303* in *Sac. erythraea*. **(A)** *SACE_0303* genome location information. **(B)** Schematic deletion of *SACE_0303* by linearized fragment homologous recombination in A226. **(C)** PCR confirmation of the *SACE_0303* deletion mutant by the primers *SACE_0303*-C1 and *SACE_0303*-C2. The size of gene 564 bp amplified by PCR bands observed in A226, 1,540 bp was detected from pUCTSR-Δ*0303* and Δ*SACE_0303*. **(D)** Er- A yields in A226, Δ*SACE_0303*, *ΔSACE_0303*/pIB139, ΔSACE_*0303*/pIB-0303, A226/pIB139, and A226/pIB-0303. **(E)** Er- A yields in WB and WB/pIB-0303. Error bars **(D)**: SD from triplicate experiments. Statistical notations **(D)**: ns, not significant; ^∗^*p* < 0.05.

The information on *SACE_0303* and its adjacent genes of the *Sac. erythraea* chromosome is shown in Figure 1A. *SACE_0304*, the neighboring gene of *SACE_0303*, encodes an MFR. To clarify the function of SACE_0303, the fragment homologous recombination method was performed in *Sac. erythraea* A226 to obtain the *SACE_0303*-deleted mutant strain Δ*SACE_0303* (Figure 1B), which was confirmed by PCR analyses (Figure 1C). The Er-A production of Δ*SACE_0303* was significantly reduced by 33% compared with A226 fermented for 6 days in the R5 liquid medium. No obvious change in cellular growth and morphological differentiation was observed between the two strains (Supplementary Figure 1). To determine that the yield decrease in Δ*SACE_0303* was only caused by inactivation of *SACE_0303*, we constructed the complement strains Δ*SACE_0303*/pIB-0303 and overexpressing strains A226/pIB-0303 based on the pIB139 vector. Results showed that pIB139 had no effect on the yield of erythromycin by testing the fermentation extracts of A226/pIB139 and Δ*SACE_0303*/pIB139 (Figure 1D) although Er-A yield of Δ*SACE_0303*/pIB-0303 was nearly recovered to the parental level, and overexpression of *SACE_0303* in A226 increased the Er-A yield by ∼31% (Figure 1D). Furthermore, we overexpressed *SACE_0303* in the high-yield *Sac. erythraea* WB and confirmed that the Er-A yield of WB/pIB-0303 was ∼25% higher than that in WB (Figure 1E). These findings indicate that *SACE_0303* has a positive effect on the biosynthesis of erythromycin.

### SACE_0303 Directly Represses *SACE_0304* but Indirectly Activates *eryAI* and *ermE*

**FIGURE 2:**
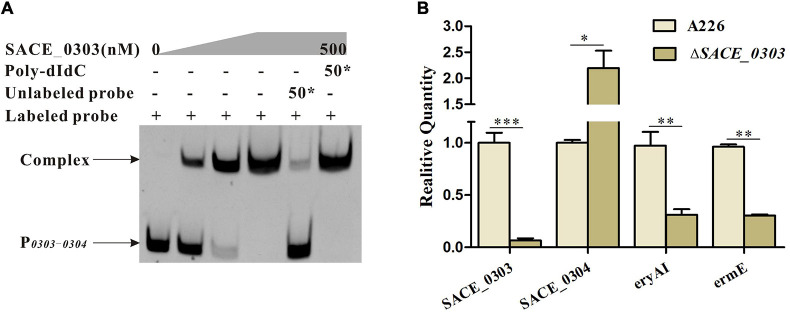
Regulatory mode of SACE_0303 in *SACE_0303* and *SACE_0304.* **(A)** EMSAs of the interaction of probe P_*0303–0304*_ with purified His_6_-SACE_0303 protein. **(B)** Effects of SACE_0303 disruption on transcripts of *SACE_0303*, *SACE_0304*, *eryAI*, and *ermE*. RT-qPCR was used to quantify the amounts of transcripts in A226 and Δ*SACE_0303* cultured for 24 h in the liquid R5 medium. Error bars **(B)**: SD from triplicate experiments. Statistical notations **(B)**: ^∗^*p* < 0.05, ^∗∗^*p* < 0.01, ^∗∗∗^*p* < 0.001.

It has been documented that actinomycete TFRs affect the biosynthesis of antibiotics by directly regulating the expression of adjacent genes (Cuthbertson and Nodwell, 2013). Thereby, EMSA was herein utilized to examine if SACE_0303 also exhibits a similar regulatory mode. The FAM-labeled probe P_*0303–0304*_ covering the entire promoter regions of *SACE_0303* and *SACE_0304* was mixed with purified His_6_-SACE_0303. After the addition of purified SACE_0303, a mobility shift was obviously observed (Figure 2A). External addition of a 50-fold excess unlabeled probe can notably compete with the labeled probe to bind to SACE_0303, while an excess 50-fold nonspecific probe, poly-dIdC, cannot abolish the shift band (Figure 2A), together providing the solid evidence that SACE_0303 bound specifically to P_*0303*–*0304*_. RT-qPCR analyses showed that *SACE_0303* was transcriptionally decreased by 93% upon its activation, but transcript of *SACE_0304* in Δ*SACE_0303* was increased by 120% (Figure 2B). These results indicate that SACE_0303 transcriptionally activates its own gene and represses *SACE_0304* via direct regulatory pattern.

Furthermore, we chose the promoter regions of erythromycin biosynthetic gene *eryAI* encoding polyketide synthase I (P*_*eryAI*_*) and the resistance gene *ermE* encoding rRNA methyltransferase (P*_*ermE*_*) for binding to His_6_-SACE_0303 to uncover its potential action mode in the *ery* cluster. Results found from the gel-shift assays that SACE_0303 could not bind to P*_*eryAI*_* and P*_*ermE*_* (Supplementary Figure 2). The transcripts of *eryAI* and *ermE* in Δ*SACE_0303* were successively reduced by 68% and 69% over those in A226 (Figure 2B). Seemingly, these data suggest that SACE_0303 exerts an indirect mode in transcriptional control of *ery* cluster.

### SACE_0304 Negatively Correlates With Erythromycin Production

**FIGURE 3:**
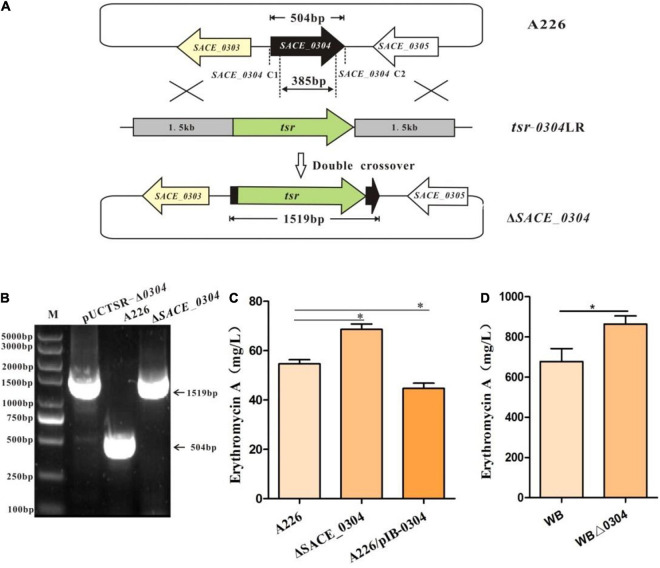
Inactivation of *SACE_0304* in *Sac. erythraea*. **(A)** Schematic deletion of *SACE_0304* by linearized fragment homologous recombination in A226. **(B)** PCR confirmation of the *SACE_0304* deletion mutant by the primers SACE_0304-C1 and SACE_0304-C2. The size of gene 504 bp amplified by PCR bands observed in A226, 1,519 bp was detected from pUCTSR-Δ*0304* and Δ*SACE_0304*. **(C)** Er-A yields in A226, Δ*SACE_0304* and A226/pIB-0304. **(D)** Er-A yields in WB and WBΔ*0304*. Error bars **(C,D)**: SD from triplicate experiments. Statistical notations **(C,D)**: ^∗^*p* < 0.05.

Because *SACE_0304* was a target gene of SACE_0303, we next examined whether it also affects erythromycin production. The Δ*SACE_0304* mutant was constructed by disrupting the *SACE_0304* gene in A226 and confirmed by PCR analyses (Figures 3A,B). Further, we performed the overexpression of *SACE_0304* under P*ermE*^∗^ in A226 to obtain the desired strain A226/pIB-0304. By HPLC analyses of cultures from those strain, we found that, compared with A226, Δ*SACE_0304* had a ∼22% production increase in Er-A, and A226/pIB-0304 exhibited a decreased yield by about 18% (Figures 3B,C). Deletion of *SACE_0304* did not affect the cell growth and morphological differentiation (Supplementary Figure 3). When *SACE_0304* was also deleted in the industrial strain WB, Er-A yield was ∼25% higher in WBΔ*0304* than of WB (Figure 3D). Thus, these results indicate that SACE_0304 has a negative effect on erythromycin production.

### SACE_0304 Directly Inhibits *SACE_0303* and *eryAI*, but Indirectly Represses *ermE*

**FIGURE 4:**
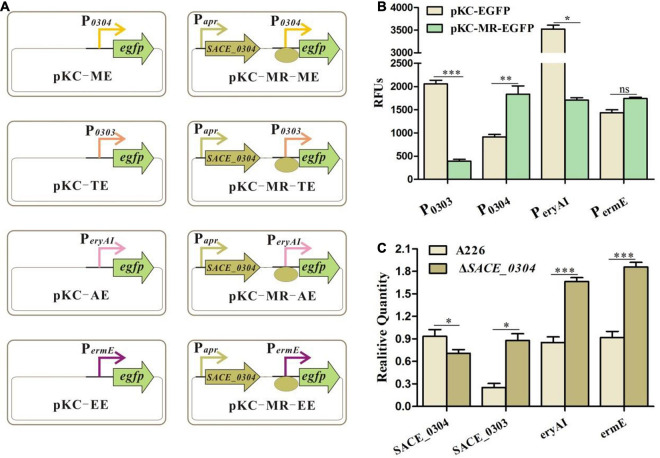
Regulatory mode of SACE_0304. **(A)** An illustration of the reporter plasmids. **(B)** Detection of the relative fluorescence units (RFUs) after different promoters in *E. coli* DH5α/pKC-EGFP and DH5α/pKC-MR-EGFP. **(C)** Effects of *SACE_0304* disruption on transcripts of *SACE_0303*, *SACE_0304*, *eryAI*, and *ermE*. Error bars **(B,C)**: SD from triplicate experiments. Statistical notations **(B,C)**: ns, not significant. ^∗^*p* < 0.05, ^∗∗^*p* < 0.01, ^∗∗∗^*p* < 0.001.

As MFRs have a similar regulatory mode as TFRs in control of their adjacent genes (Romero-Rodriguez et al., 2015), we first performed EMSA with purified SACE_0304 protein for binding to the probe P_*0303–0304*_, but this protein seemed inactive with considerable repeats. Then, we designed the eGFP reporter system in *E. coli* to verify the interaction of SACE_0304 with the promoter regions of *SACE_0303* (P_*0303*_) and *SACE_0304* (P_*0304*_) (Figure 4A). Plasmids pKC-TE and pKC-ME containing *egfp* under control of P_*0303*_ and P_*0304*_ were successively constructed and transformed into *E. coli* DH5α, and obtained strains exhibited obvious fluorescent signals. When *SACE_0304* under the promoter of apramycin resistance gene (P*_*apr*_*) was individually ligated into pKC-TE and pKC-ME and transformed into DH5α, the green fluorescence of pKC-MR-TE and pKC-MR-ME was, respectively, decreased by 81% and increased twofold compared with the absence of SACE_0304 (Figure 4B), indicating that SACE_0304 directly repressed P_*0304*_ and activated P_*0303*_ in the heterologous *E. coli* host. Further, we used RT-qPCR to compare the transcriptional levels of *SACE_0303* and *SACE_0304* between A226 and Δ*SACE_0304*. Compared with A226, Δ*SACE_0304* exhibited upregulated transcription of *SACE_0303* by 2.5-fold but downregulated transcription of *SACE_0304* by 25% (Figure 4C). Therefore, these *in vivo* and *in vitro* results demonstrate that SACE_0304 is directly self-activated and represses the expression of *SACE_0303*.

To further investigate the regulatory mode of SACE_0304 in the *ery* cluster, we likewise constructed eGFP reporter plasmids (Figure 4A), which were introduced to DH5α. As shown in Figure 4B, the fluorescence of *egfp* initiated by P*_*eryAI*_* was reduced by 50% after the addition of SACE_0304. When *SACE_0304* under P*_*apr*_* was inserted into the pKC-EE, the fluorescence almost had no statistical difference compared with that without *SACE_0304* (Figure 4B). RT-qPCR analyses showed that the expressional levels of *eryAI* and *ermE* in Δ*SACE_0304* was onefold higher than those in A226 (Figure 4C). These findings indicate that SACE_0304 directly inhibits *eryAI* but indirectly represses *ermE*.

### Determination of Precise DNA-Binding Site of SACE_0303

**FIGURE 5:**
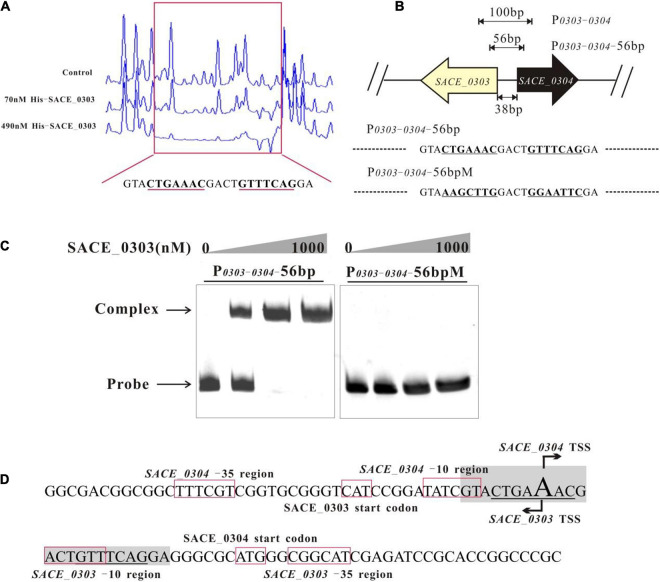
Determination of the SACE_0303-binding sites. **(A)** DNase I footprinting assay of SACE_0303-binding site within P_*0303–0304*_. Upper fluorogram: control reaction without protein. Protection regions were acquired with increasing concentrations (70 and 490 nM) of His6- SACE_0303. **(B)** Different probes designed for determining DNA binding site of SACE_0303. **(C)** Binding assays of SACE_0303 to P_*0303–0304*_-*56* bp and P_*0303–0304*_-*56* bpM. **(D)** Nucleotide sequences of promoter regions of *SACE_0303* and *SACE_0304*. Transcriptional start site, TSS.

To determine the DNA binding site of SACE_0303, a DNase I footprinting assay was manipulated using purified His_6_-SACE_0303 and the 100 bp probe P_*0303–0304*_ labeled via FAM/HEX, and results showed that a 23-nt sequence (GTACTGAAACGACTGTTTCAGGA) was protected by SACE_0303, in which an 18 bp palindrome sequence (CTGAAACGACTGTTTCAG, underlined) was obviously detected (Figures 5A,B). To verify the indispensable palindrome sequence for binding SACE_0303, we introduced mutations to repeat motifs in P_*0303–0304*_-56 bp to obtain the probe P_*0303–0304*_-56 bpM for EMSAs (Figure 5B). Results found that SACE_0303 only bound to P_*0303–0304*_-56 bp (Figure 5C). With individual prediction of the -10/-35 regions and transcriptional start sites (TSSs) of *SACE_0303* and *SACE_0304* by online software phiSITE^1^ and BDGB^2^, the repeat motifs were found to both overlap the putative −10 regions and TSSs of *SACE_0303* and *SACE_0304* (Figure 5D). Possibly, SACE_0303 represses *SACE_0304* by impeding the recruitment of RNA polymerase. The mechanism concerning self-activation of SACE_0303 needs to be further explored due to the unusualness of binding of transcriptional activator to −10 region.

### Identification and Characterization of New SACE_0303 Targets

**FIGURE 6:**
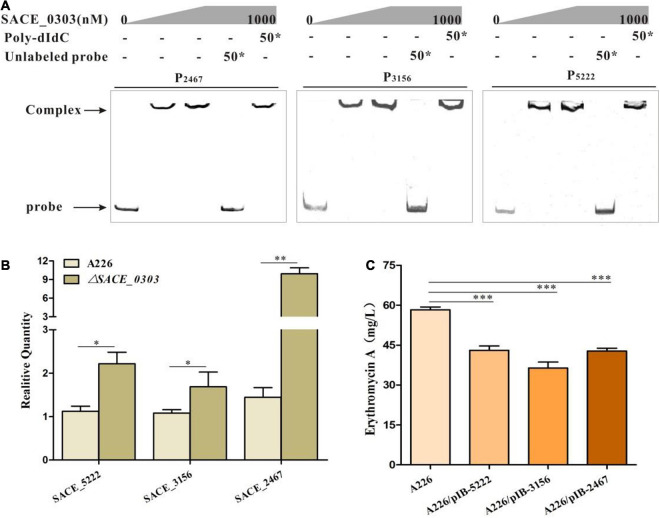
Effects of SACE_0303 on its three targets *SACE_2467*, *SACE_3156*, and *SACE_5222*. **(A)** Binding assays of SACE_0303 to P_2467_, P_3156_, and P_5222_. **(B)** Transcriptional analyses of *SACE_2467*, *SACE_3156*, and *SACE_5222* in A226 and Δ*SACE_0303.* **(C)** Er-A yields in A226, A226/pIB-2467, A226/pIB-3156, and A226/pIB-5222. Error bars **(B,C)**: SD from triplicate experiments. Statistical notations **(B,C)**: *p < 0.05, **p < 0.01, ***p < 0.001.

Uncovering the regulatory network of SACE_0303 requires the identification of additional SACE_0303 target genes. To this end, we used the above 18 bp SACE_0303-binding sequences to scan the whole genome sequence of *Sac. erythraea* by an online virtual footprint software suite^3^. Totally, the upstream regions of 83 genes containing P_*0303–0304*_ were identified (cutoff score ≥20) (data not shown), during which 10 predicted high-score sites flanked by well-annotated genes were chosen for EMSAs (Supplementary Table 4). Results confirm that SACE_0303 bound specifically to the promoter regions of *SACE_2467* encoding cation-transporting ATPase (P_*2467*_), *SACE_3156* encoding a large transcriptional regulator (P_*3156*_), and *SACE_5222* encoding alpha-ketoglutarate permease (P_*5222*_) (Figure 6A). Further RT-qPCR analyses showed that the transcripts of these three genes were increased in Δ*SACE_0303* to varying degrees compared with those in A226 (Figure 6B). These results demonstrate that SACE_0303 directly controls the transcription of *SACE_2467*, *SACE_3156*, and *SACE_5222*.

To uncover the effects of the three genes on the erythromycin production, *SACE_2467*, *SACE_3156*, and *SACE_5222* were individually overexpressed in A226. By fermentation and HPLC analyses, Er-A yields in A226/pIB-2467, A226/pIB-3156, and A226/pIB-5222 were, respectively, reduced by 26.6, 37.6, and 26.1% relative to those in A226 (Figure 6C), indicating that these three target genes of SACE_0303 were negatively correlated with erythromycin production.

### Integrative Engineering Toward SACE_0303-Mediated Mini-Regulatory Network in a High-Yield Strain

Given that *SACE_0303* overexpression or *SACE_0304* deletion in WB both enhanced the Er-A yield (Figures 1E, 3D), we further performed multiplex engineering of the two TFs as well as three new SACE_0303 target genes to estimate their practical potential in the high-yield strain of *Sac. erythraea*.

**FIGURE 7:**
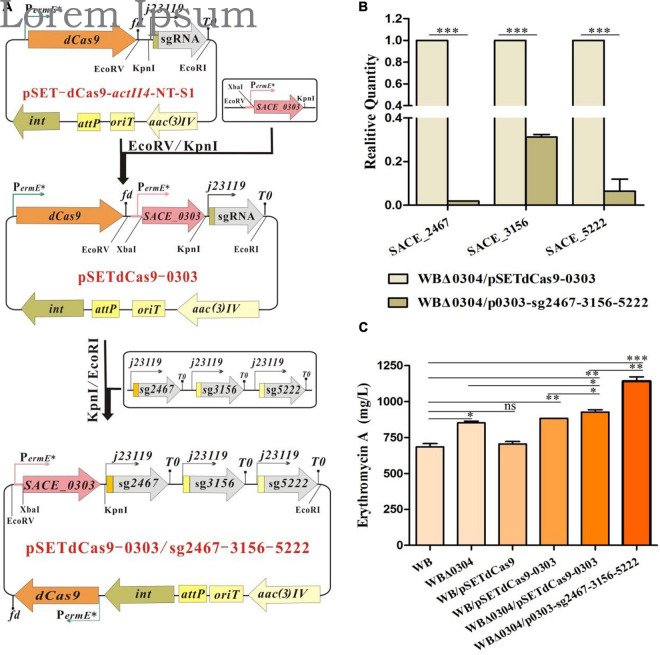
Multiplex engineering of SACE_0303-mediated regulatory system in WB. **(A)** Schematic construction of pSET-dCas9*-*0303/sg2467-3156-5222. **(B)** Transcriptional assays of *SACE_2467*, *SACE_3156*, and *SACE_5222* in WBΔ*0304*/pSETdCas9-0303 and WBΔ*0304*/p0303-sg2467-3156-5222. **(C)** Er-A yield in WB and its derived mutants. Error bars **(B,C)**: SD from triplicate experiments. Statistical notations **(B,C)**: ns, not significant; ^∗^*p* < 0.05, ^∗∗^*p* < 0.01, ^∗∗∗^*p* < 0.001.

To use the CRISPRi system for multigene repression, pSET-dCas9 (Figure 7A), a pSET152-derived integrative plasmid (Zhao et al., 2018) was transformed into WB to evaluate its effect on erythromycin production. Results showed that the Er-A yield of WB/pSETdCas9 was not significantly different from that of WB (Figure 7C). In pSET-dCas9-*actII4*-NT-S1 with available digestion sites to ligate sgRNA-containing cassette, *SACE_0303* under P*_*ermE*_^∗^* was ligated into the CRISPRi vector to generate recombinant plasmid pSETdCas9-0303 (Figure 7A), which was then introduced into WB and WBΔ*0304*, respectively. These resulting strains WB/pSETdCas9-0303 (884 mg/L) and WBΔ*0304*/pSETdCas9-0303 (928 mg/L) exhibited a stepwise increase in Er-A yield over WB (685 mg/L) (Figure 7C). Furthermore, a synthetic cassette containing the sgRNAs of *SACE_2467*, *SACE_3156*, and *SACE_5222* was ligated into pSETdCas9-0303 (Figure 7A and Supplementary Figure 4), and the desired plasmid pSET-dCas9*-*0303/sg2467-3156-5222 was transferred into WBΔ*0304* to generate the strain WBΔ*0304*/p0303-sg2467-3156-5222. As expected, the transcriptional levels of *SACE_2467*, *SACE_3156*, and *SACE_5222* in WBΔ*0304*/p0303-sg2467-3156-5222 were inhibited to different degrees via RT-qPCR analyses (Figure 7B). Correspondingly, WBΔ*0304*/p0303-sg2467-3156-5222 (1,142 mg/L), respectively, exhibited ∼23% and ∼67% increase in Er-A production compared with WBΔ*0304*/pSETdCas9-0303 and WB (Figure 7C).

## Discussion

Up to now, five TFRs from *Sac. erythraea* were successively shown to be involved in the repression or activation of erythromycin biosynthesis, in which SACE_7301 and SACE_3446 exerted a direct interaction to the promoters of the *ery* cluster (Wu et al., 2014a, 2016), and SACE_3986 and SACE_5754 indirectly controlled the transcription of the *ery* cluster (Wu et al., 2014b, 2019). However, the regulatory network regarding erythromycin biosynthesis has been seldom reported; in particular, hierarchical regulations between TFRs and other TFs have not been investigated yet. In this study, we report a novel TFR, SACE_0303, and reveal reciprocal regulation between this TFR and its adjoining MFR SACE_0304. With the DNA-binding site of SACE_0303 defined, three new SACE_0303 target genes were identified and confirmed to repress the erythromycin production. By integrating CRISPRi-mediated repression of the three targets with *SACE_0304* deletion and *SACE_0303* overexpression, combinatorial engineering of this mini-regulatory network was performed in a high-yield strain, resulting in dramatic titer improvement of erythromycin.

TFRs generally serve as repressors to transcriptionally regulate their upstream targets for the control of antibiotic production (Deng et al., 2013; Xu et al., 2019a). A similar phenomenon was also found in *Sac. erythraea*, in which four of the hitherto reported TFRs played the negative role in regulation of erythromycin biosynthesis (Wu et al., 2014b, 2016, 2019; Xu et al., 2018). Only the TFR, SACE_7301, was shown to trigger the transcription of *eryAI* and *ermE* by interacting with their promoter regions for positively regulating the erythromycin production (Wu et al., 2014a). In this study, SACE_0303 was likewise affirmed to positively correlate with erythromycin production (Figure 1); however, it indirectly stimulated the transcription of *eryAI* and *ermE* (Figure 2), implicating a SACE_0303-mediated regulation for the erythromycin biosynthesis via hierarchical control pattern. Then, SACE_0303 was demonstrated to directly inhibit *SACE_0304* (Figure 2). SACE_0304, negatively affecting erythromycin production (Figure 3), was proved, in turn, to directly repress *SACE_0303* as well as *eryAI* but indirectly inhibit *ermE* (Figure 4). Accordingly, our findings exhibit a very complicated mechanism for cascaded control of erythromycin production by the two types of TFs.

Members of the multiple antibiotic resistance regulators (MarR) family of TFs, widely distributing among prokaryotes, could modulate diverse physiological processes, including stress response, antibiotic resistance, and export, etc. (Grove, 2013). In spite of abundant distribution of MFRs in the antibiotic-producing actinomycetes, just a few members have been characterized in *Streptomyces* (Yang et al., 2010; Davis et al., 2013; Huang and Grove, 2013; Zhang et al., 2015; Guo et al., 2018; Kong et al., 2019; Nag and Mehra, 2021), and the functional probe into MFRs in *Sac. erythraea* has not been reported. Until lately, we identified an MFR (SACE_6745) to directly inhibit the genes for erythromycin biosynthesis, export, and resistance (Liu et al., 2021a). This study identified an additional MFR (SACE_0304) in *Sac. erythraea* and proved its direct transcriptional control of erythromycin production. It is believed that, together with SACE_6745, the identification of SACE_0304 provides the starting point to deepen the understanding of MFR-mediated regulation for erythromycin biosynthesis.

TFRs and MFRs, both serving as one-component regulators, could control the expression of upstream targets by responding to specific ligands (Cuthbertson and Nodwell, 2013; Grove, 2013). Typically, they could bind to intergenic regions to autoregulate their own gene and divergently transcribed gene (Romero-Rodriguez et al., 2015). We herein found that SACE_0303 and SACE_0304 indeed abided by the paradigm that they are both self-activated directly and can repress the expression of each other (Figures 2, 4). Whether certain or common ligands exist to affect reciprocal regulation of the two TFs needs to be further explored.

Based on defined DNA binding site of SACE_0303 within P_*0303–0304*_ (Figure 5), we utilized genome scanning, EMSAs, and transcriptional assays to identify three new SACE_0303 target genes, *SACE_2467*, *SACE_5222*, and *SACE_3156*, which were next proved to negatively affect erythromycin production (Figure 6). SACE_2467 encodes a P-type ATPase, which could generally utilize energy released by ATP hydrolysis to transport cations across the cell membrane (Sitsel et al., 2015). A previous report shows that deletion of SCO2731 (a P-type ATPase) and its adjacent SCO2730 (a copper chaperon) activated secondary metabolic pathways in *S. coelicolor* by enabling cytosolic copper to optimal homeostasis (Gonzalez-Quinonez et al., 2019). As the erythromycin biosynthesis was recently shown to positively correlate with the ATP/ADP ratio in *Sac. erythraea* (Li et al., 2020), we inferred that *SACE_2467* overexpression might consume more ATP to transport cations, subsequently decreasing erythromycin production. α-ketoglutarate (α-KG), an intermediate of the tricarboxylic acid (TCA) cycle, intersects between carbon and nitrogen metabolic pathways (Commichau et al., 2006). In *S. coelicolor*, increased α-KG could promote the TCA cycle to form more NADH for maintaining intracellular redox homeostasis (Xu et al., 2017). We speculate that overexpression of *SACE_5222* encoding α-ketoglutarate permease might unbalance intracellular redox status, exhibiting an adverse effect on erythromycin biosynthesis. SACE_3156 encodes a large transcriptional regulator belonging to a LuxR family, and its overexpression likewise decreased the erythromycin yield (Figure 6). However, the regulatory mechanism of SACE_3156 requires further investigation. Building on current results, the SACE_0303-mediated mini-regulatory network was proposed (Supplementary Figure 5), which not only controls different types of TFs to exert the regulatory cascade, but also might affect cofactors hemostasis and metabolism for control of erythromycin production.

Rewiring the regulatory network with engineering of TFs and their targets is an effective approach to boost the productivity of antibiotics in actinomycetes (Martín and Liras, 2010; Xia et al., 2020). For example, certain TFs and (or) their targets have been manipulated in *Sac. erythraea* WB for enhanced erythromycin production (Wu et al., 2014a,b, 2016, 2019; Liu et al., 2017, 2019). Nevertheless, few genes were jointly manipulated, and two genes at most were hitherto knocked out simultaneously in the high-yield strain (Wu et al., 2016; Liu et al., 2021a). Herein, we manage to exert stepwise engineering of three types of TFs and two metabolic genes in WB. Specifically, *SACE_0303* under P*ermE*^∗^ was ligated into the CRISPRi system and the obtained plasmid was introduced into an existing WB mutant with *SACE_0304* deletion for concurrent transcriptional downregulation of three new SACE_0303 targets as well as *SACE_0303* overexpression (Figure 7). Expectedly, corresponding mutants displayed stepwise titer improvement, in which a final strain with joint engineering of five genes had ∼67% increase in Er-A yield over WB (Figure 7). The present study provides a new tactic for antibiotic yield improvement by TF-based combinatorial engineering of industrial actinomycetes.

## Data Availability Statement

The raw data supporting the conclusions of this article will be made available by the authors, without undue reservation.

## Author Contributions

HW and BZ conceived and designed the study. YL, SK, PW, BL, LL, JN, HZ, and KC performed the experiments. YL and HW analyzed the data. HW wrote the manuscript. BZ modified the manuscript. All authors have read and approved the manuscript.

## Conflict of Interest

The authors declare that the research was conducted in the absence of any commercial or financial relationships that could be construed as a potential conflict of interest.

## Publisher’s Note

All claims expressed in this article are solely those of the authors and do not necessarily represent those of their affiliated organizations, or those of the publisher, the editors and the reviewers. Any product that may be evaluated in this article, or claim that may be made by its manufacturer, is not guaranteed or endorsed by the publisher.

## Footnotes

1
http://www.phisite.org/

2
http://www.fruitfly.org/

3
http://www.prodoric.de/vfp/

## Supplementary Material

Supplementary Figure 1 Morphological differentiation and growth rates of A226 and Δ*SACE_0303*.

Supplementary Figure 2 EMSAs of DNA-binding of SACE_0303 to P*_*eryAI*_* and P*_*ermE*_*.

Supplementary Figure 3 Morphological differentiation and growth rates of A226 and Δ*SACE_0304*.

Supplementary Figure 4 The synthetic cassette of sg*2467-3156-5222*.

Supplementary Figure 5 Possible regulatory pathway of SACE_0303 for control of erythromycin biosynthesis in *Sac. erythraea*.

Supplementary Table 1 Strains used in this study.

Supplementary Table 2 Plasmids used in this study.

Supplementary Table 3 Primers used in this study.

Supplementary Table 4 EMSAs for upstream regions of putative SACE_0303 target genes.

## References

[B1] Barka E. A. Vatsa P. Sanchez L. Gaveau-Vaillant N. Jacquard C. Meier-Kolthoff J. P. (2016). Taxonomy, physiology, and natural products of actinobacteria. *Microbiol. Mol. Biol. Rev.* 80 1–43. 10.1128/MMBR.00019-15 26609051 PMC4711186

[B2] Chng C. Lum A. M. Vroom J. A. Kao C. M. (2008). A key developmental regulator controls the synthesis of the antibiotic erythromycin in Saccharopolyspora erythraea. *Proc. Natl. Acad. Sci. U.S.A.* 105 11346–11351. 10.1073/pnas.0803622105 18685110 PMC2516264

[B3] Commichau F. M. Forchhammer K. Stulke J. (2006). Regulatory links between carbon and nitrogen metabolism. *Curr. Opin. Microbiol.* 9 167–172. 10.1016/j.mib.2006.01.001 16458044

[B4] Cuthbertson L. Nodwell J. R. (2013). The TetR family of regulators. *Microbiol. Mol. Biol. Rev.* 77 440–475. 10.1128/MMBR.00018-13 24006471 PMC3811609

[B5] Davis J. R. Brown B. L. Page R. Sello J. K. (2013). Study of PcaV from *Streptomyces coelicolor* yields new insights into ligand-responsive MarR family transcription factors. *Nucleic Acids Res.* 41 3888–3900. 10.1093/nar/gkt009 23396446 PMC3616709

[B6] Deng W. Li C. Xie J. (2013). The underling mechanism of bacterial TetR/AcrR family transcriptional repressors. *Cell Signal* 25 1608–1613. 10.1016/j.cellsig.2013.04.003 23602932

[B7] Gonzalez-Quinonez N. Corte-Rodriguez M. Alvarez-Fernandez-Garcia R. Rioseras B. Lopez-Garcia M. T. Fernandez-Garcia G. (2019). Cytosolic copper is a major modulator of germination, development and secondary metabolism in *Streptomyces coelicolor*. *Sci. Rep.* 9:4214. 10.1038/s41598-019-40876-0 30862861 PMC6414726

[B8] Grove A. (2013). MarR family transcription factors. *Curr. Biol.* 23 R142–R143. 10.1016/j.cub.2013.01.013 23428319

[B9] Guo J. Zhang X. Lu X. Liu W. Chen Z. Li J. (2018). SAV4189, a MarR-family regulator in *Streptomyces avermitilis*, activates avermectin biosynthesis. *Front. Microbiol.* 9:1358. 10.3389/fmicb.2018.01358 30013524 PMC6036246

[B10] Han S. Song P. Ren T. Huang X. Cao C. Zhang B. (2011). Identification of SACE_7040, a member of TetR family related to the morphological differentiation of *Saccharopolyspora erythraea*. *Curr. Microbiol.* 63 121–125. 10.1007/s00284-011-9943-z 21626147

[B11] Hellman L. M. Fried M. G. (2007). Electrophoretic mobility shift assay (EMSA) for detecting protein–nucleic acid interactions. *Nature Protocols* 2 1849–1861. 10.1038/nprot.2007.249 17703195 PMC2757439

[B12] Huang H. Grove A. (2013). The transcriptional regulator TamR from *Streptomyces coelicolor* controls a key step in central metabolism during oxidative stress. *Mol. Microbiol.* 87 1151–1166. 10.1111/mmi.12156 23320788

[B13] Kong L. Liu J. Zheng X. Deng Z. You D. (2019). CtcS, a MarR family regulator, regulates chlortetracycline biosynthesis. *BMC Microbiol.* 19:279. 10.1186/s12866-019-1670-9 31823730 PMC6905112

[B14] Li X. Chen J. Andersen J. M. Chu J. Jensen P. R. (2020). Cofactor engineering redirects secondary metabolism and enhances erythromycin production in *Saccharopolyspora erythraea*. *ACS Synth. Biol.* 9 655–670. 10.1021/acssynbio.9b00528 32078772

[B15] Liu J. Chen Y. Li L. Yang E. Wang Y. Wu H. (2019). Characterization and engineering of the Lrp/AsnC family regulator SACE_5717 for erythromycin overproduction in *Saccharopolyspora erythraea*. *J. Ind. Microbiol. Biotechnol.* 46 1013–1024. 10.1007/s10295-019-02178-2 31016583

[B16] Liu J. Chen Y. Wang W. Ren M. Wu P. Wang Y. (2017). Engineering of an Lrp family regulator SACE_Lrp improves erythromycin production in *Saccharopolyspora erythraea*. *Metab. Eng.* 39 29–37. 10.1016/j.ymben.2016.10.012 27794466

[B17] Liu J. Li L. Wang Y. Li B. Cai X. Tang L. (2021a). Joint engineering of SACE_Lrp and its target MarR enhances the biosynthesis and export of erythromycin in *Saccharopolyspora erythraea*. *Appl. Microbiol. Biotechnol.* 105 2911–2924. 10.1007/s00253-021-11228-8 33760930

[B18] Liu Y. Wang H. Li S. Zhang Y. Cheng X. Xiang W. (2021b). Engineering of primary metabolic pathways for titer improvement of milbemycins in *Streptomyces bingchenggensis*. *Appl. Microbiol. Biotechnol.* 105 1875–1887. 10.1007/s00253-021-11164-7 33564920

[B19] Martín J. F. Liras P. (2010). Engineering of regulatory cascades and networks controlling antibiotic biosynthesis in *Streptomyces*. *Curr. Opin. Microbiol.* 13 263–273. 10.1016/j.mib.2010.02.008 20303823

[B20] Mironov V. A. Sergienko O. V. Nastasiak I. N. Danilenko V. N. (2004). Biogenesis and regulation of biosynthesis of erythromycins in *Saccharopolyspora erythraea*: a review. *Prikl. Biokhim. Mikrobiol.* 40 613–624. 15609849

[B21] Nag A. Mehra S. (2021). A major facilitator superfamily (MFS) efflux pump, SCO4121, from *Streptomyces coelicolor* with roles in multidrug resistance and oxidative stress tolerance and its regulation by a MarR regulator. *Appl. Environ. Microbiol.* 87:e02238-20. 10.1128/AEM.02238-20 33483304 PMC8091613

[B22] Niu G. Chater K. F. Tian Y. Zhang J. Tan H. (2016). Specialised metabolites regulating antibiotic biosynthesis in *Streptomyces* spp. *FEMS Microbiol. Rev.* 40 554–573. 10.1093/femsre/fuw012 27288284

[B23] Oliynyk M. Samborskyy M. Lester J. B. Mironenko T. Scott N. Dickens S. (2007). Complete genome sequence of the erythromycin-producing bacterium *Saccharopolyspora erythraea* NRRL23338. *Nat. Biotechnol.* 25:447. 10.1038/nbt1297 17369815

[B24] Robertsen H. L. Musiol-Kroll E. M. (2019). Actinomycete-derived polyketides as a source of antibiotics and lead structures for the development of new antimicrobial drugs. *Antibiotics (Basel)* 8:157. 10.3390/antibiotics8040157 31547063 PMC6963833

[B25] Romero-Rodriguez A. Robledo-Casados I. Sanchez S. (2015). An overview on transcriptional regulators in *Streptomyces*. *Biochim. Biophys. Acta* 1849 1017–1039. 10.1016/j.bbagrm.2015.06.007 26093238

[B26] Sitsel O. Grønberg C. Autzen H. E. Wang K. Meloni G. Nissen P. (2015). Structure and function of Cu(I)- and Zn(II)-AT pases. *Biochemistry* 54 5673–5683. 10.1021/acs.biochem.5b00512 26132333

[B27] Tian J. Yang G. Gu Y. Sun X. Lu Y. Jiang W. (2020). Developing an endogenous quorum-sensing based CRISPRi circuit for autonomous and tunable dynamic regulation of multiple targets in *Streptomyces*. *Nucleic Acids Res.* 48 8188–8202. 10.1093/nar/gkaa602 32672817 PMC7430639

[B28] van der Heul H. U. Bilyk B. L. McDowall K. J. Seipke R. F. van Wezel G. P. (2018). Regulation of antibiotic production in actinobacteria: new perspectives from the post-genomic era. *Nat. Prod. Rep.* 35 575–604. 10.1039/c8np00012c 29721572

[B29] Wilkinson C. J. Hughes-Thomas Z. A. Martin C. J. Bohm I. Mironenko T. Deacon M. (2002). Increasing the efficiency of heterologous promoters in actinomycetes. *J. Mol. Microbiol. Biotechnol.* 4 417–426. 12125822

[B30] Wu H. Chen M. Mao Y. Li W. Liu J. Huang X. (2014a). Dissecting and engineering of the TetR family regulator SACE_7301 for enhanced erythromycin production in *Saccharopolyspora erythraea*. *Microb. Cell Fact.* 13 1–11. 25391994 10.1186/s12934-014-0158-4 PMC4258057

[B31] Wu H. Chu Z. Zhang W. Zhang C. Ni J. Fang H. (2019). Transcriptome-guided target identification of the TetR-like regulator SACE_5754 and engineered overproduction of erythromycin in *Saccharopolyspora erythraea*. *J. Biol. Eng.* 13:11. 30697347 10.1186/s13036-018-0135-2 PMC6346578

[B32] Wu H. Wang Y. Yuan L. Mao Y. Wang W. Zhu L. (2016). Inactivation of SACE_3446, a TetR family transcriptional regulator, stimulates erythromycin production in *Saccharopolyspora erythraea*. *Synth. Syst. Biotechnol.* 1 39–46. 10.1016/j.synbio.2016.01.004 29062926 PMC5640589

[B33] Wu P. Pan H. Zhang C. Wu H. Yuan L. Huang X. (2014b). SACE_3986, a TetR family transcriptional regulator, negatively controls erythromycin biosynthesis in *Saccharopolyspora erythraea*. *J. Ind. Microbiol. Biotechnol.* 41 1159–1167. 10.1007/s10295-014-1449-9 24793123

[B34] Xia H. Li X. Li Z. Zhan X. Mao X. Li Y. (2020). The application of regulatory cascades in streptomyces: yield enhancement and metabolite mining. *Front. Microbiol.* 11:406. 10.3389/fmicb.2020.00406 32265866 PMC7105598

[B35] Xie S. Shen B. Zhang C. Huang X. Zhang Y. (2014). sgRNAcas9: a software package for designing CRISPR sgRNA and evaluating potential off-target cleavage sites. *PLoS One* 9:e100448. 10.1371/journal.pone.0100448 24956386 PMC4067335

[B36] Xu F. Wang J. Zhao G. P. (2017). Alpha-ketoglutarate protects *Streptomyces coelicolor* from visible light-induced phototoxicity. *Biochem. Biophys. Rep.* 9 22–28. 10.1016/j.bbrep.2016.11.002 29114580 PMC5632709

[B37] Xu Y. Ke M. Li J. Tang Y. Wang N. Tan G. (2019a). TetR-type regulator SLCG_2919 is a negative regulator of lincomycin biosynthesis in *Streptomyces lincolnensis*. *Appl. Environ. Microbiol.* 85:e02091-18. 10.1128/AEM.02091-18 30341075 PMC6293104

[B38] Xu Y. You D. Yao L. L. Chu X. Ye B. C. (2019b). Phosphate regulator PhoP directly and indirectly controls transcription of the erythromycin biosynthesis genes in *Saccharopolyspora erythraea*. *Microb. Cell Fact.* 18:206. 10.1186/s12934-019-1258-y 31775761 PMC6880422

[B39] Xu Z. Liu Y. Ye B.-C. (2018). PccD regulates branched-chain amino acid degradation and exerts a negative effect on erythromycin production in *Saccharopolyspora erythraea*. *Appl. Environ. Microbiol.* 84:e00049-18. 29439982 10.1128/AEM.00049-18 PMC5881055

[B40] Yang Y. H. Song E. Lee B. R. Kim E. J. Park S. H. Kim Y. G. (2010). Rapid functional screening of *Streptomyces coelicolor* regulators by use of a pH indicator and application to the MarR-like regulator AbsC. *Appl. Environ. Microbiol.* 76 3645–3656. 10.1128/AEM.02617-09 20382814 PMC2876474

[B41] Zhang Q. Chen Q. Zhuang S. Chen Z. Wen Y. Li J. (2015). A MarR family transcriptional regulator, DptR3, activates daptomycin biosynthesis and morphological differentiation in *Streptomyces roseosporus*. *Appl. Environ. Microbiol.* 81 3753–3765. 10.1128/AEM.00057-15 25819953 PMC4421045

[B42] Zhao Y. Li L. Zheng G. Jiang W. Deng Z. Wang Z. (2018). CRISPR/dCas9-mediated multiplex gene repression in *Streptomyces*. *Biotechnol. J.* 13:e1800121. 10.1002/biot.201800121 29862648

