# Supplementary material for: Uncovering and Engineering a Mini-Regulatory Network of the TetR-Family Regulator SACE_0303 for Yield Improvement of Erythromycin in Saccharopolyspora erythraea
Source: Front Bioeng Biotechnol. 2021 Sep 14;9:692901. doi: 10.3389/fbioe.2021.692901 (PMC8476842; doi:10.3389/fbioe.2021.692901)
Supplement: Supplementary Figure 1 — Morphological differentiation and growth rates of A226 and ΔSACE_0303. [file Presentation_1.pdf]

Supplementary Materials

Table S1 Strains used in this study

Strains	Relevant characteristic	Source or reference
E. coli		
DH5α	f *recA lac*ZM15	Invitrogen
BL21 (DE3)	F-*ompThsd*SB (*rB*$^-$*mB*$^-$) *gal dcm* (DE3)	Novagen
Saccharopolyspora erythraea		
A226	An erythromycin low producer	CGMCC 8279
Δ*SACE_0303*	A226 with *SACE_0303* deleted	This study
Δ*SACE_0303*/pIB139	Δ*SACE_0303* carrying pIB139	This study
Δ*SACE_0303*/pIB-*0303*	Δ*SACE_0303* carrying pIB139-*0303*	This study
A226/pIB139	A226 carrying pIB139	This study
A226/pIB-*0303*	A226 carrying pIB139-*0303*	This study
Δ*SACE_0304*	A226 with *SACE_0304* deleted	This study
A226/pIB-*0304*	A226 carrying pIB139-*0304*	This study
A226/pIB-*2467*	A226 carrying pIB139-*2467*	This study
A226/pIB-*3156*	A226 carrying *p*IB139-*3156*	This study
A226/pIB-*5222*	A226 carrying *p*IB139-*5222*	This study
WB	An erythromycin overproducer	CGMCC 8280
WBΔ*SACE_0304*	WB with *SACE_0304* deleted	This study
WBpSETdCas9	WB carrying pSETdCas9	This study
WB/pSETdCas9-*0303*	WB carrying pSETdCas9-0303	This study
WBΔ*0304*/pSETdCas9-*0303*	WBΔ*SACE_0304* carrying pSETdCas9-0303	This study
WBΔ*0304*/p*0303*-sg*2467-3156-5222*	WBΔ*SACE_0304* carrying pSETdCas9-*0303*-sg*2467-3156-5222*	This study

Table S2 Plasmids used in this study

**Plasmids**	**Relevant characteristic**	**Source or reference**
pUCTSR	pUC18 derivative containing a 1.36-kb fragment of a thiostrepton resistance gene	(Wu et al. 2016)
pUCTSR-Δ*0303*	pUCTSR derivative containing two 1.5-kb fragments, the upstream and downstream regions of *SACE_0303*	This study
pUCTSR-Δ*0304*	pUCTSR derivative containing two 1.5-kb fragments adjoining *SACE_0304*	This study
pIB139	C31 *attP-int* locus, *aac(3)IV*, *oriT*, *ermE** promoter	(Wilkinson et al. 2002)
pIB139-*0303*	pIB139 derivative for expression of *SACE_0303*	This study
pIB139-*0304*	pIB139 derivative for expression of *SACE_0304*	This study
pIB139-*2467*	pIB139 derivative for expression of *SACE_2467*	This study
pIB139-*3156*	pIB139 derivative for expression of *SACE_3156*	This study
pIB139-*5222*	pIB139 derivative for expression of *SACE_5222*	This study
pSETdCas9	C31 *attP-int* locus, *aac(3)IV*, *oriT*, *ermE** promoter, dCas9	(Zhao et al. 2018)
pSETdCas9-*actII4*-NT-S1	pSETdCas9 derivative carrying sgRNA of *actII4*	(Zhao et al. 2018)
pUC-*0303*	pUC18 derivative carrying P*ermE** for expression of *SACE_0303*	This study
pSETdCas9-*0303*	pSETdCas9-*actII4*-NT-S1 derivative carrying P*ermE** for expression of *SACE_0303*	This study
pUC57-sg2467-3156-5222	pUC57 derivative carrying sgRNA of *SACE_2467*, *SACE_3156*, *SACE_5222*	This study
pSETdCas9-0303-sg2467-3156-5222	pSETdCas9-0303 derivative carrying sgRNA of *SACE_2467*, *SACE_3156*, *SACE_5222*	This study
pET28a	His tag, *kan*, T7 promoter	Novagen
pET28a-*0303*	pET28b derivative carrying *SACE_0303*	This study
pKC1139	*aac(3)IV*, pSG5 *ori, PK2 oriT, ermE** promoter	(Wilkinson et al. 2002)
pKC-AE	pKC1139 derivative carrying P_{eryAI} for expression of *egfp*	This study
pKC-MR-AE	pKC-AE derivative carrying $P_{aac(3)IV}$ for expression of *SACE_0304*	This study
pKC-EE	pKC1139 derivative carrying P_{ermE} for expression of *egfp*	This study
pKC-MR-EE	pKC-EE derivative carrying $P_{aac(3)IV}$ for expression of *SACE_0304*	This study
pKC-TE	pKC1139 derivative carrying P_{0303} for expression of *egfp*	This study
pKC-MR-TE	pKC-TE derivative carrying $P_{aac(3)IV}$ for expression of *SACE_0304*	This study
pKC-ME	pKC1139 derivative carrying P_{0304} for expression of *egfp*	This study
pKC-MR-ME	pKC-ME derivative carrying $P_{aac(3)IV}$ for expression of *SACE_0304*	This study

Table S3 Primers used in this study

**name**	**Sequence (5'-3')**<br>**(restriction site underlined)**	**use**
SACE_0303 down -F	AAAGGTACCGCGCTACGCGGCCTGATGCT (*Kpn*I)	Deletion of *SACE_0303* gene
SACE_0303 down -R	AAAGAATTCTCAGCGGGTAGTCGCCGTGC (*Eco*RI)	
SACE_0303 up - F	CCCAAGCTTGGCGGGTCAACTTCGGGCAC (*Hin*dIII)	
SACE_0303 up -R	CCCTCTAGAGCCGTGGTCGGCCGAGTACT (*Xba*I)	
SACE_0303-C1	AAACATATGATGACCCGCACCGACGAAAG (*Nde*I)	Complementation and overexpression of *SACE_0303*
SACE_0303-C2	AAATCTAGACTACGCCGGCTCGACCGGCA (*Xba*I)	
SACE_0304 up - F	CCCAAGCTTCCGGGGTGTTGTTGCCGACG (*Hin*dIII)	Deletion of *SACE_0304* gene
SACE_0304 up -R	CCCTCTAGACAGCACGGTGCCCAGGTCTC (*Xba*I)	
SACE_0304 down -F	AAAGAGCTCGCCCGGCTCTACACCGAAGC (*Sac*I)	
SACE_0304 down -R	AAAGAATTCCTACATGGCGGTCGGCGGCA (*Eco*RI)	
SACE_0304-C1	AAACATATGATGGGCGGCATCGAGATCCG (*Nde*I)	Complementation and overexpression of *SACE_0304*
SACE_0304-C2	AAATCTAGACTAGAGGTCGAGGTCGTAAC (*Xba*I)	
SACE_2467-C1	CGCCATATGGTGACCACAGCCACTCCTG (*Nde*I)	Overexpression of *SACE_2467*
SACE_2467-C2	TGCTCTAGATCAGCGGGTGGCCGTG (*Xba*I)	
SACE_3156-C1	CGCCATATGATGCCCACGTCCGCG (*Nde*I)	Overexpression of *SACE_3156*
SACE_3156-C2	TGCTCTAGATCAACTCCACCGCCGTACCC (*Xba*I)	
SACE_5222-C1	CGCCATATGGTGGGCCTGCGCA (*Nde*I)	Overexpression of *SACE_5222*
SACE_5222-C2	TGCTCTAGAATCGAAGCCAAGCTCTCAGTGG (*Xba*I)	
Apr-test-F	GGAGTGCATATGGTGCAATACGAATGGCGAAAAG	Confirmation of *S. erythraea* mutants containing pIB139, pSETdCas9 and their derived plasmids.
Apr-test-R	CTCAAAGCTTCAGCCAATCGACTGGCGAGCG	
PermE*-F	CCCAAGCTTGATATCCTCTAGTATGCATGCGAG (*Eco*RV)	Amplification of *ermE** promoter
PermE*-R	TGCTCTAGAATGTGGATCCTACCAACCGGC (*Xba*I)	
SACE_0303-C3	TGCTCTAGAATGACCCGCACCGACGAAAGC (*Xba*I)	Overexpression of *SACE_0303*

SACE_0303-C4	CGGGGTACCCTACGCCGGCTCGACC (*Kpn*I)	
SACE_0303-C5	AAACATATGATGACCCGCACCGACGAAAG（*Nde*I）	Expression and purification of SACE_0303
SACE_0303-C6	CCCAAGCTTCTACGCCGGCTCGACCGGCA（*Hin*dIII）	
P0303-F (F)	CCCAAGCTTGCGGGCCGGTGC (*Hin*dIII)	Amplification of *SACE_0303* promoter
P0303-R (F)	CCCTCTAGAGGCGACGGCGGCTTT (*Xba*I)	
P0304-F (F)	CCCAAGCTTAGTACTCCAGCAGCTTGGACAGCAG (*Hin*dIII)	Amplification of *SACE_0304* promoter
P0304-R (F)	GGGTCTAGAGCGCCCTCCTGAAACAGTCGTTTC (*Xba*I)	
PeryAI-F (F)	CCCAAGCTTGAATCACTGATCCCATTCACCGGAGCAT (*Hin*dIII)	Amplification of *eryAI* promoter
PeryAI-R (F)	GGGTCTAGAAGCTTTGACAGGTCCGCCACGCG (*Xba*I)	
PermE-F (F)	CCCAAGCTTGCGAGTGTCCGTTCGAGTGGCGG (*Hin*dIII)	Amplification of *ermE* promoter
PermE-R (F)	GGGTCTAGACGCTGGATCCTACCAACCGGCAC (*Xba*I)	
P-Papr-F (F)	AAAGATATCGACGCTCAGTGGAACGAA (*Eco*RV)	Amplification of *aac(3)IV* promoter
P-Papr-R (F)	AAACATAGAGACATTGCACTCCACCGCT (*Nde*I)	
SACE_0304-F (F)	AAACATATGATGGGCGGCATCGAGATCCG (*Nde*I)	Expression of SACE_0304
SACE_0304-R (F)	AAAGAATTCCTAGAGGTCGAGGTCGTAACGGGC (*Eco*RI)	
PeryAI-F	GAATCACTGATCCCATTCACCGGAGCAT	Probe P_{eryAI}
PeryAI-R	AGCTTTGACAGGTCCGCCACGCG	
PermE-F	GCGAGTGTCCGTTCGAGTGGCGG	Probe P_{ermE}
PermE-R	CGCTGGATCCTACCAACCGGCAC	
SACE_0303-SACE_0304-F	GGCGACGGCGGCTTTCGTCG	Probe *0303-0304*-int
SACE_0303-SACE_0304-R	GCGGGCCGGTGCGGATCTCG	
P0303-0304-F	GGCGACGGCGGCTTTCGTCG	Probe *0303-0304*-int with FAM
P0303-0304-R	GCGGGCCGGTGCGGATCTCG	Probe *0303-0304*-int with HEX
0303-0304-56bp-F	GGGTCATCCGGATATCGTA	Probe *0303-0304*-56bp
0303-0304-56bp-R	ATGCCGCCCATGCGCCCTC	

T-0303-0304-F	GGGTCATCCGGATATCGTAAAGCTTGGACTGGAATTC GAGGGCGCATGGGCGGCAT	Probe *0303-0304*-56bpM
T-0303-0304-R	ATGCCGCCCATGCGCCCTCGAATTCCAGTCCAAGCTTT ACGATATCCGGATGACCC	
P1030-F	GGCCGCTGGGAAGGTGATCGGG	Probe P_{1030}
P1030-R	CGGCAGTACCGGGTCGTCTTCCCC	
P2167-F	GCTACCTGTGCCGTCGGATGCCAG	Probe P_{2167}
P2167-R	GCTCGCCCTCCAGGGTCCGTGC	
P2467-F	CCACACCCCGCTGGCACCAACT	Probe P_{2467}
P2467-R	CAGGTCACCTCGTCGCGCGCG	
P2891-F	CCAGTCCCCACTTTCATTCCGATCCTG	Probe P_{2891}
P2891-R	GTTGGTCTCTATCTCCAGGTCACGGCC	
P3156-F	CGACCTCGGCCGCCTGCTGT	Probe P_{3156}
P3156-R	CGAGGACGTGTTCTGCCGGGCG	
P3603-F	GGCCGCACCGCACCCGGT	Probe P_{3603}
P3603-R	GCCGGGCACGGTAGCGGCCTTC	
P4008-F	GGCGCACCTCCTCGATTGTTCGGC	Probe P_{4008}
P4008-R	CGTCGACCTCCCTCGGTTCGGGTG	
P5066-F	CCCCGCAGGCAGTTGCTGGACC	Probe P_{5066}
P5066-R	GGCTCAACCAACCGTTCGTTTGCACTAGG	
P5222-F	CCGGCGTCCACTGAGCAACGCG	Probe P_{5222}
P5222-R	GCCAAACCTCCACGTTGAGGTGCTGATG	
P5626-F	CACTTCGGCCTCCAGGTTGTGTGCCG	Probe P_{5626}
P5626-R	GGTCAGCCTCCGAGTCCACCACTCCG	
eryAI-RT-F	GACCTGTCAAAGCTCTCCGA	RT-qPCR analysis of *eryAI*
eryAI-RT-R	GATCGAGATGTGCACGCAAT	
ermE-RT-F	GAGTGGGAGTTCGTCGAGA	RT-qPCR analysis of *ermE*
ermE-RT-R	ACCATCGACTCGTAGCGTTC	
0304-RT-F	CTCCGCTGGTTCCTGGTC	RT-qPCR analysis of *SACE_0304*

0304-RT-R	CCACCGTCCAGAGCTTGA	
0303-RT-F	CCTGATGCTCGACCTGCT	RT-qPCR analysis of *SACE_0303*
0303-RT-R	ACCAGTGACTCGAAGCCC	
2467-RT-F	TGCAACATGGCTTACTCAGG	RT-qPCR analysis of *SACE_2467*
2467-RT-R	GCTAGCTTCTGCGTCAAAGG	
3156-RT-F	GGTCGTGGTCGAGGACAT	RT-qPCR analysis of *SACE_3156*
3156-RT-R	GTAGGTGACCACGACCCAGA	
5222-RT-F	CAACTGCTGGCGATCATCTA	RT-qPCR analysis of *SACE_5222*
5222-RT-R	ACAGCATACCGACCAGAACC	

Table S4 EMSAs for the predicted target genes of SACE_0303

Synonym	product	Scores	sequence
SACE_2467	cation-transporting ATPase	25.26	CTGAAACGAGCGGTGAAG
SACE_5222	alpha-ketoglutarate permease	24.65	CTGAAGCGGCCGTTTCCA
SACE_1030	leucine aminopeptidase	23.48	CTGAACCGAATCTTGAGG
SACE_3603	aminoglycoside acetyltransferase	22.87	CTGAACCGGGGGTTTTGG
SACE_5626	XRE family transcriptional regulator	21.7	CTGAACGGAGTGGTGGAC
SACE_2167	regulatory protein MarR	21.09	CTGAAAAGATAGGCGAAG
SACE_2891	TetR family transcriptional regulator	21.09	CTGAACGGAACGTTATCG
SACE_3156	large transcriptional regulator	21.09	CTGAAACGGTTGCGAAAA
SACE_5066	protein kinase/LuxR family transcriptional regulator	21.09	CTGAATTGCCTAGTGCAA
SACE_0633	citrate synthase	20.53	CTGAAAAAAGGTTGTAAA
SACE_4008	3-oxoadipate:succinyl-CoA transferase subunit A	20.53	CTGAATTCAGTATTGGAC

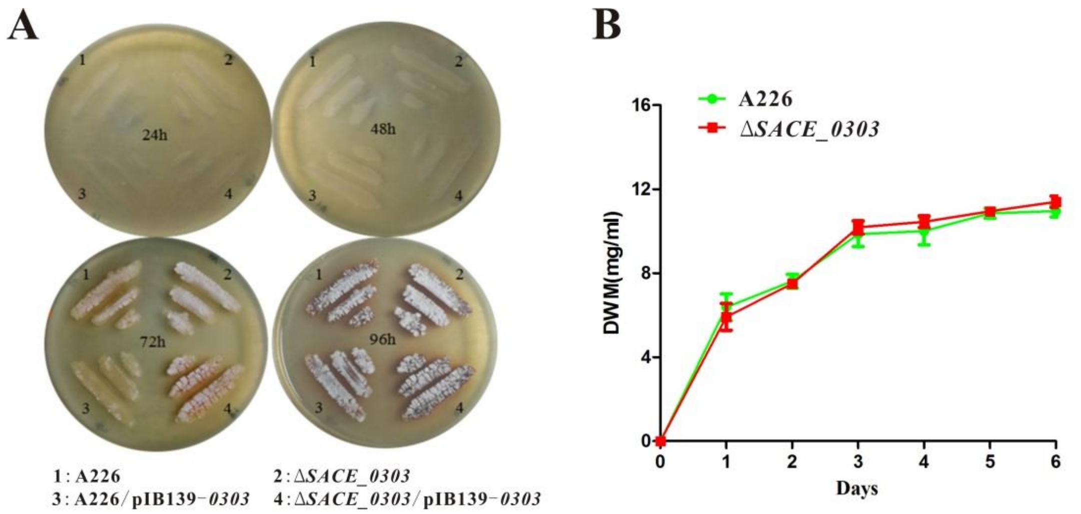

Figure S1 Morphological differentiation and growth rates of A226 and Δ*SACE_0303*. (A) Aerial mycelia formation of A226, Δ*SACE_0303*, A226/pIB139-*0303*. The four strains were grown on R3M agar medium at 30℃ for 24, 48, 72 and 96 h. (B) Growth curves of A226 and Δ*SACE_0303*. The two strains were cultured in the R5 liquid medium, and their dry weights of mycelia (DWM) were measured.

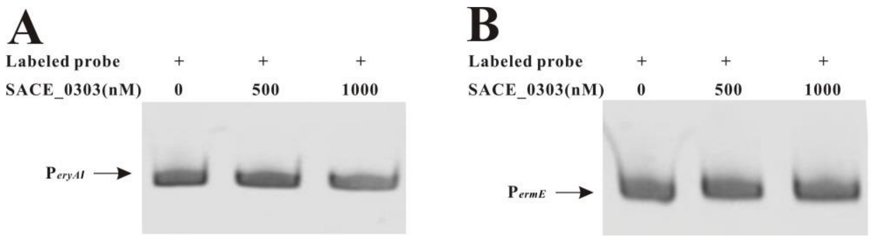

Figure S2 EMSAs of DNA-binding of SACE_0303 to P*eryAI* and P*ermE*. (A) EMSAs of DNA-binding of SACE_0303 to P*eryAI*. (B) EMSAs of DNA-binding of SACE_0303 to P*ermE*.

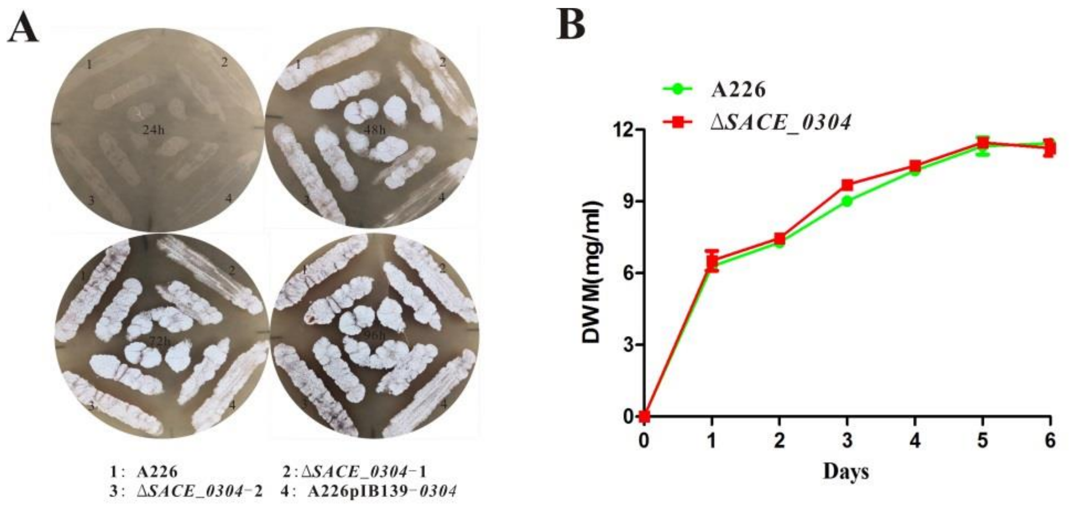

Figure S3 Morphological differentiation and growth rates of A226 and Δ*SACE_0304*. (A) Aerial mycelia formation of A226, Δ*SACE_0304*-1, Δ*SACE_0304*-2 and A226/pIB139-*0304*. The four strains were grown on R3M agar medium at 30℃ for 24 h, 48 h, 72 h and 96 h. (B) Growth curves of A226 and Δ*SACE_0304*. The two strains were cultured in the R5 liquid medium, and their dry weights of mycelia (DWM) were measured.

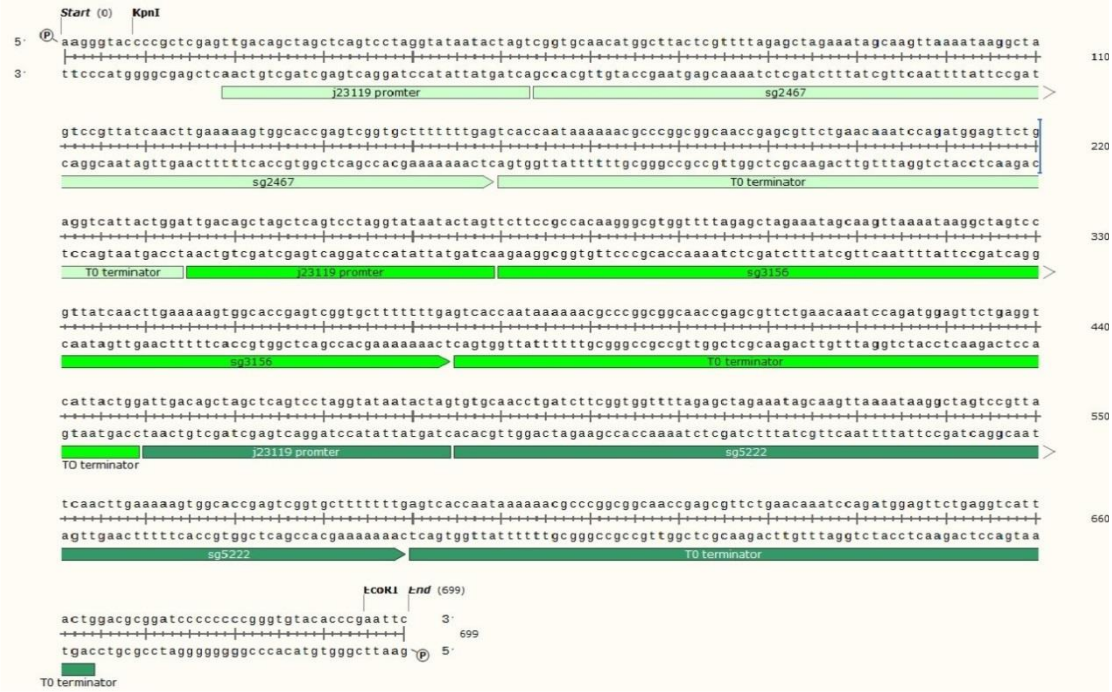

Figure S4 The synthetic cassette of sg*2467-3156-5222*.

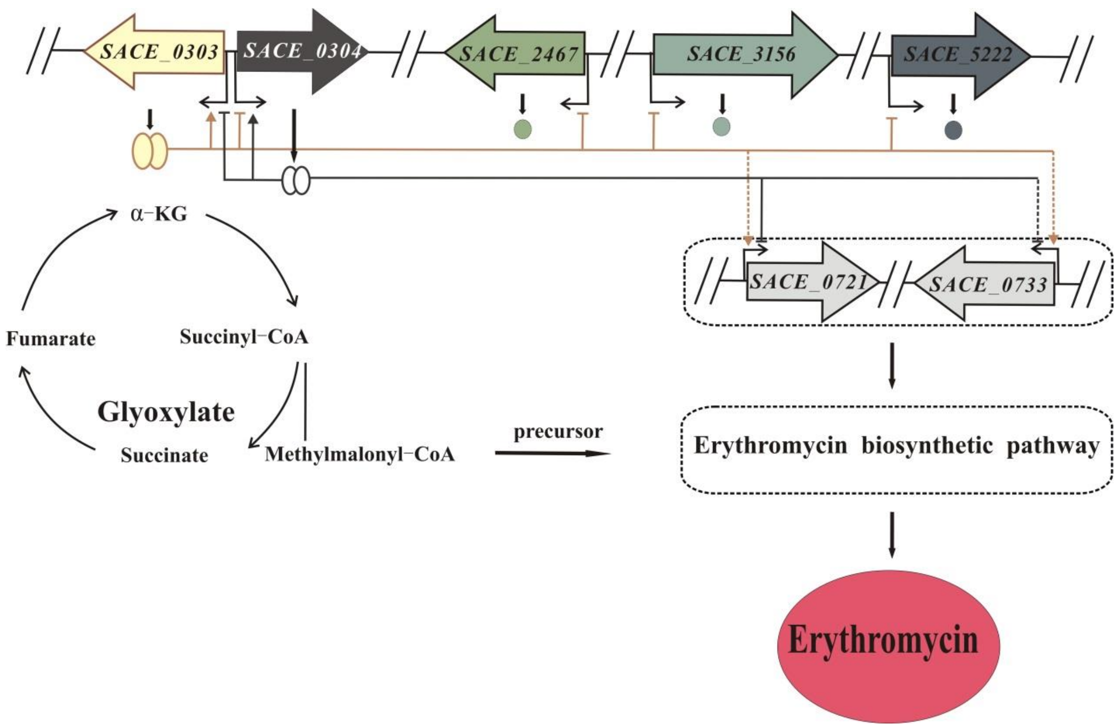

Figure S5 Probability regulatory pathways of SACE_0303 for control of erythromycin biosynthesis in *Sac. erythraea*.

References

Wilkinson CJ, Hughes-Thomas ZA, Martin CJ, Bohm I, Mironenko T, Deacon M, Wheatcroft M, Wirtz G, Staunton J, Leadlay PF (2002) Increasing the efficiency of heterologous promoters in actinomycetes. J Mol Microbiol Biotechnol 4(4):417-26

Wu H, Wang Y, Yuan L, Mao Y, Wang W, Zhu L, Wu P, Fu C, Müller R, Weaver DT, Zhang L, Zhang B (2016) Inactivation of SACE_3446, a TetR family transcriptional regulator, stimulates erythromycin production in *Saccharopolyspora erythraea*. Synth Syst Biotechnol 1(1):39-46 doi:10.1016/j.synbio.2016.01.004

Zhao Y, Li L, Zheng G, Jiang W, Deng Z, Wang Z, Lu Y (2018) CRISPR/dCas9-Mediated Multiplex Gene Repression in *Streptomyces*. Biotechnol J 13(9):e1800121 doi:10.1002/biot.201800121
